# Gifting future scientists the past through well-preserved specimens of modern microbial ecosystems

**DOI:** 10.1038/s41467-025-62138-6

**Published:** 2025-07-19

**Authors:** A. Murat Eren

**Affiliations:** 1 Helmholtz Institute for Functional Marine Biodiversity, Oldenburg, Germany; 2 Alfred Wegener Institute, Helmholtz Centre for Polar and Marine Research, Bremerhaven, Germany; 3 Institute for Chemistry and Biology of the Marine Environment, University of Oldenburg, Oldenburg, Germany; 4 Marine Biological Laboratory, Woods Hole, MA USA; 5 Max Planck Institute for Marine Microbiology, Bremen, Germany

**Keywords:** Microbiology, Environmental sciences, Scientific community

## Abstract

Historical specimens have enabled transformative insights across kingdoms and ecosystems with new technologies, yet microbes remain largely overlooked in preservation efforts. As we recognize microbial communities as fundamental drivers of planetary health, comprehensive microbial archiving becomes an urgent intergenerational responsibility.

## Main

Trying to piece together life’s history on our planet has been one of the most fundamental endeavors in life sciences. From Aristotle’s Scala Naturae to Ernst Haeckel’s intricate illustrations, from Charles Darwin’s theory of evolution to Carl Woese’s molecular reconstructions, countless efforts spanning millennia have sought to elucidate life by characterizing its distinct entities, describing processes that maintain its diversity, and understanding mechanisms by which life initiates and responds to planetary change.

The search for answers to fundamental scientific questions has always been catalyzed by advances in technology. As tools and algorithms improve, so does our desire to revisit historical samples, compare them to their modern analogs, and identify the factors that ultimately determine whether the entities of life persist or disappear in their ever-changing environments. Since time travel will likely remain forever off the table in our universe^1^, our best chance to analyze high-quality and well-characterized samples from the past with future technologies is to archive the present.

A powerful demonstration of the importance of archival efforts comes from modern museums and herbaria. By providing researchers with access to well-preserved specimens of now-vanished individuals and ecosystems, these archives have enabled insights that would otherwise remain out of reach. For instance, using herbarium specimens that spanned over 200 years, Woodward showed that rising atmospheric CO₂ led to significant physiological changes in plants^2^; Lang et al. later uncovered the genetic basis of this response, revealing that plants not only respond with short-term physiological adjustments, but also evolve through adaptive genetic processes to cope with human-driven environmental change^3^. Using historical specimens from 1350, Bos et al. reconstructed a *Yersinia pestis* genome from victims of the Black Death^4^, and demonstrated that medieval and modern populations of *Y. pestis* do not dramatically differ from one another, indicating that the nature of the disease today is determined by social, medical, and ecological practices rather than the genetics of the causative agent itself. Using museum samples dating back to 1871, Murray et al. showed that extinction of the passenger pigeon, once the most abundant bird in North America, was likely a function of evolutionary processes reshaping the population genome to keep up with environmental perturbations^5^, revealing that even species with large and stable population sizes are not immune to extinction following rapid changes in their environment. These examples and many others demonstrate the power of biological archives in reconstructing life histories and applying their lessons to the present. Yet despite these successes, a critical gap remains as archival efforts have largely focused on plant and animal specimens, overlooking the microbial residents of our host-associated and other natural habitats.

Given how recent our recognition of the existence of microbes and their incomprehensible diversity is, it is not surprising that they have not historically attracted the same attention as plants and animals in archival efforts. But as a result, modern surveys of microbial systems are unable to take advantage of samples collected centuries ago with their microbial contents in mind, and applications of advanced technologies to study complex microbial systems largely remain limited to contemporary samples that cover relatively short time frames. As we begin to recognize microbial life as a fundamental driver and indicator of planetary health^6^, and learn how to unlock the vast potential of microbes to address key challenges in health, environmental sustainability, and basic science^7^, the need to address this archival gap becomes increasingly urgent.

Multiple researchers have advocated for the need to prioritize long-term preservation of microbiomes associated with humans and plants^8,9^, and recent initiatives seek to address the omission of microbes in archival samples. Two examples of such initiatives include the Global Microbiome Conservancy (GMbC, https://microbiomeconservancy.org/), which aims to establish a biobank of human gut microbial isolates from diverse populations around the globe, and the UK Crop Microbiome CryoBank (UK-CMCB, https://www.cabi.org/projects/the-uk-crop-microbiome-cryobank/), which aims to secure microbial communities from crops that are critical for agriculture. Most recently, the Microbiota Vault Initiative (MVI, https://www.microbiotavault.org/) joined these global biobanking efforts, with the goal of preserving microbial samples for future study, and ‘safeguarding microbial diversity’, particularly of the human-associated microbiota^10^. MVI’s approach involves standardized collection, cryopreservation, and storage of microbial samples to enable future resuscitation and analysis, and the initiative strives to combine its technical infrastructure with a global governance model considers benefit-sharing, inclusivity, and ethical stewardship^10^.

Our planet is facing formidable challenges that are exacerbated by climate change, including a rapid loss of biodiversity^11^. Whether the great depletion that has afflicted plants and animals has also occurred among microbes is not yet clear, but numerous observations have revealed a reduction in microbial diversity that is linked to industrialized lifestyle at least for the human gut environment^12^, which is also associated with a dramatic increase in human autoimmune disorders^13^. Given these interlinked crises, it is understandable that all prominent microbial archiving efforts target microbes that are directly associated with human wellbeing, through isolates or fecal samples from the human gut, or microbial isolates from plants or fermented foods that are key to human food security. Similar to other efforts, the MVI pilot phase starts with a focus on human-associated microbes. But the MVI differs from previous efforts as a standalone, non-profit entity substantiated by a comprehensive feasibility study that considers technical, organizational, legal, ethical, and economical aspects of implementing a long-term strategy from sample collection to storage to access (https://www.microbiotavault.org/), and its promise to broaden archival efforts to environmental samples.

It remains uncertain whether we can counteract the ongoing loss of biodiversity by resuscitating extinct organisms, and whether such organisms could survive without continuous human intervention. Nevertheless, such attempts will always attract attention. Not only because ‘bringing something back’ offers a nostalgic allure, but also because it represents a profound exercise of power: the ability to decide what is to be summoned into existence and on what terms. In parallel, it is critical to recognize that our basic science, archival attempts, or efforts to resuscitate what is lost are unlikely to have lasting impact without transformative societal change that addresses the human drivers of biodiversity loss^14^.

Luckily, we can pass such practical concerns to future generations, along with well-preserved samples that represent our time. In that quest, incorporating microbes in our historical archives is a fundamental responsibility as it is the only way to enable future scientists who will study ecological, environmental, and health priorities of their time with the latest technological advances to peer into the past. Scientists, policymakers, and funding agencies must support innovative, practical archival efforts to ensure future generations a robust, viable record of Earth’s microbial heritage. Ideally, one that captures not only human-associated microbes but also those associated with other animals and plants, aquatic systems, soils, and sediments.

## References

[1] Hawking, S. W. Chronology protection conjecture. *Phys. Rev. D. Part. Fields* **46**, 603–611 (1992). 10014972 10.1103/physrevd.46.603

[2] Woodward, F. I. Stomatal numbers are sensitive to increases in CO2 from pre-industrial levels. *Nature* **327**, 617–618 (1987).

[3] Lang, P. L. M. et al. Century-long timelines of herbarium genomes predict plant stomatal response to climate change. *Nat. Ecol. Evol.* **8**, 1641–1653 (2024). 39117952 10.1038/s41559-024-02481-x PMC11383800

[4] Bos, K. I. et al. A draft genome of Yersinia pestis from victims of the Black Death. *Nature* **478**, 506–510 (2011). 21993626 10.1038/nature10549 PMC3690193

[5] Murray, G. G. R. et al. Natural selection shaped the rise and fall of passenger pigeon genomic diversity. *Science* **358**, 951–954 (2017). 29146814 10.1126/science.aao0960

[6] Crowther, T. W. et al. Scientists’ call to action: Microbes, planetary health, and the Sustainable Development Goals. *Cell* **187**, 5195–5216 (2024). 39303686 10.1016/j.cell.2024.07.051

[7] Eren, A. M. & Banfield, J. F. Modern microbiology: Embracing complexity through integration across scales. *Cell* **187**, 5151–5170 (2024). 39303684 10.1016/j.cell.2024.08.028 PMC11450119

[8] Bello, M. G. D., Knight, R., Gilbert, J. A. & Blaser, M. J. Preserving microbial diversity. *Science* **362**, 33–34 (2018). 30287652 10.1126/science.aau8816

[9] Raaijmakers, J. M. & Kiers, E. T. Rewilding plant microbiomes. *Science* **378**, 599–600 (2022). 36356130 10.1126/science.abn6350

[10] Dominguez-Bello, M. G. et al. The microbiota vault initiative: safeguarding Earth’s microbial heritage for future generations. *Nat. Commun.* **16**, 5373 (2025). 40579417 10.1038/s41467-025-61008-5 PMC12205078

[11] Pörtner, H.-O. et al. Overcoming the coupled climate and biodiversity crises and their societal impacts. *Science* **380**, eabl4881 (2023). 37079687 10.1126/science.abl4881

[12] Sprockett, D. D. & Coyte, K. Z. When microbes go missing: Understanding the impact of diversity loss within the gut microbiome. *Cell Host Microbe* **31**, 1249–1251 (2023). 37562357 10.1016/j.chom.2023.07.006

[13] Miller, F. W. The increasing prevalence of autoimmunity and autoimmune diseases: an urgent call to action for improved understanding, diagnosis, treatment, and prevention. *Curr. Opin. Immunol.* **80**, 102266 (2023). 36446151 10.1016/j.coi.2022.102266 PMC9918670

[14] O’Brien, K. et al. IPBES Transformative Change Assessment: Summary for policymakers. Preprint at 10.5281/ZENODO.11382230 (2025).

